# The prospect of carbon fiber implants in radiotherapy

**DOI:** 10.1120/jacmp.v13i4.3821

**Published:** 2012-07-05

**Authors:** Ni Xin‐ye, Tang Xiao‐bin, Geng Chang‐ran, Chen Da

**Affiliations:** ^1^ College of Material Science and Technology Nanjing University of Aeronautics and Astronautics Nanjing 210016 China

**Keywords:** carbon fiber implants, radiotherapy, dose calculation, Monte Carlo

## Abstract

Because of their superior characteristics, carbonaceous materials, which are still at their early stage of development, have garnered significant interest. Because of their low atomic number, carbonaceous orthopedic implants possess radiation properties similar to biological tissues and, therefore, they are more suitable to patients in need of radiotherapy. The effects of stainless steel, titanium, and carbon plates on radiation dose distributions were investigated in this work using Monte Carlo simulations and TLD measurements for 6 MV photon beams. It is found that carbon plates will neither increase the incident surface dose, nor lead to the decrease of exit surface dose (the effect of a second build‐up). Carbon fiber orthopedic implants have a good prospect for radiotherapy patients because they have minimal perturbation effects on the radiotherapy dose distribution.

PACS number: 87.55.K‐,87.55.Gh, 87.55.ne

## I. INTRODUCTION

Traditional orthopedic implant materials are usually stainless steel or titanium whose atomic numbers are larger than those of normal tissues in bodies. In radiotherapy, these metal implants significantly differ from human normal tissue in density, and hence will perturb the radiation dose distribution in the body.^(^ ^1^ ^)^ In contrast, carbonaceous materials have low atomic numbers, good biocompatibility, chemical stability, good mechanical properties, and modulus of elasticity similar to human bones.^(^ ^2^ ^–^ ^5^ ^)^ The availability of carbon implants will solve the problem of dose perturbation for traditional metal implants. This is good news for those cancer patients who may receive radiotherapy treatments after implanting orthopedic implants.^(^ ^6^ ^)^

There are strengths and shortcomings for all artificial implants. For example, metals have the disadvantages of electrolysis, fatigue, wear, corrosion, loosening, and bone absorption. Polymer materials have the problems of aging, poor creep resistance, and toxic reactions. Ceramic materials have no plasticity, and are crisp and easy to break. In recent years, carbonaceous materials have garnered significant medical interest, and are being investigated for clinical applications because of their good biological and chemical stability, good mechanical properties, and modulus of elasticity similar to human bone. The carbon fiber implants investigated in this work are carbon–carbon composite materials rather than traditional carbon–resin composite materials, because carbon–carbon composite materials are not toxic to normal tissues.^(^ ^7^ ^)^ However, carbon fiber implants are not as good as metals in terms of malleability and impact toughness. Under great pressure, the carbon fiber may fracture and the implants may rupture. Meister et al.^(^ ^8^ ^)^ and Pryor et al.^(^ ^9^ ^)^ found that with wear and tear the tissues surrounding carbonaceous implants could be dyed black, and the surrounding cells may contain carbon particles. These shortcomings may be solved by, for example, wrapping a layer of metal and thick carbon biological capacitive materials around the carbon fiber implants to prevent the release of carbon particles.^(^ ^7^ ^,^ ^10^ ^)^

In this work, we investigated the perturbation effects of stainless steel implants, titanium implants, and carbon fiber implants on radiotherapy dose distributions. We computed dose distributions using commercial treatment planning systems, and performed Monte Carlo simulations and measurements for stainless steel, titanium alloy, and carbon materials using 6 MV photon beams. The Monte Carlo method is the most accurate method to calculate radiation dose distributions in the body^(^ ^11^ ^,^ ^12^ ^)^ that cannot be measured using currently available radiation detectors.

## II. MATERIALS AND METHODS

### A.1 Orthopedic implants investigated

**Figure 1:**
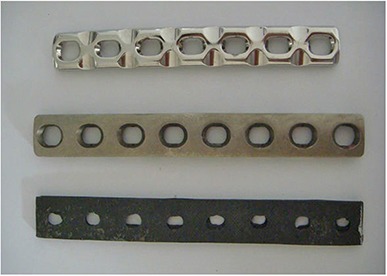
Orthopedic implants of different materials (from top to bottom: stainless steel, titanium alloy, and carbon fiber).

We investigated three types of orthopedic implants in this work (Fig. 1). Stainless steel implants consist of iron, chromium, nickel, and a small amount of nitrogen, manganese, silicon, sulfur, and molybdenum. Stainless steel implants have good mechanical properties and are easily corroded in the body, so they are often used as temporary implants. Titanium alloy implants contain aluminum (Al) and vanadium (V) with good corrosion resistance. The carbon fiber implants were made of three‐dimensional PAN‐based carbon–carbon composite materials (The College of Material Science and Technology, Nanjing University of Aeronautics and Astronautics, Nanjing, China). These three types of implants are 0.4 cm in thickness, 1.3, 1.5, and 1.5 cm in width, and 12.5, 14, and 14 cm in length, respectively. Their mass densities are 7.91, 4.54, and $1.7\;g/{\text{cm}}^{3}$, respectively.

### A.2 Monte Carlo simulations

**Figure 2:**
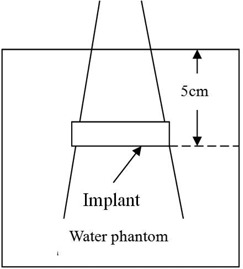
The experimental setup for the implant irradiation.

In this work, the BEAMnrc Monte Carlo code was used to simulate the 6 MV photon beams of a Siemens PRIMUS linear accelerator (Siemens AG, Erlangen, Germany). The Monte Carlo simulation source was the No. 0 source (a parallel beam from the front). The radius of the source was 0.5 mm. The total number of source particles simulated was $1\times 10^{9}$ to ensure that the $1_{-\sigma }$ statistical uncertainty of the dose distributions was less than 2%. BEAMnrc generated phase space data files (egsphsp), which were 2–4 GB in size. The DOSXYZnrc Monte Carlo code was used for the dose calculation. The field size was 10 cm × 10 cm and 3 cm × 3 cm, respectively, defined at a source‐to‐surface distance (SSD) of 100 cm. The implants were centered at 5 cm depth in a 30 cm×30 cm×50 cm water phantom. The cutoff energy was 0.7 MeV (total energy) for electron transport and 0.01 MeV (kinetic energy) for photon transport, in both accelerator simulation and phantom dose calculation. The maximum electron step length was set to 5 cm, and the computational grid size was 0.1 cm×0.1 cm×0.1 cm (Fig. 2). The implant geometries used for the Monte Carlo simulation were represented by the three‐dimensional rectilinear geometry of the simulation phantom based on the actual implants in Fig. 1.

### A.3 TLD measurements in a water phantom

TLD measurements were performed in water for 6 MV photon beams with a 10 cm × 10 cm field defined at 100 cm SSD. To ensure the accuracy of the measurements, TLD chips were prescreened by repeated radiation at 200–500 cGy and only those chips showing small measurement uncertainties (within 4%) were selected. To compare the dose changes with and without implants, three TLD chips were placed at the same locations above and below the implants and irradiated with 200 monitor units (MU). The experiments were repeated three times and the results were analyzed.

### A.4 X‐ray and CT imaging of implants implanted in animals

The stainless steel, titanium, and carbon fiber implants were implanted in the same thigh of a pig, and fixed with carbon fiber screws. The whole animal was scanned on a Nucletron SIMULIX HQ simulator (Nucletron, Veenendaal, The Netherlands) and a Siemens SOMATOM CT scanner.

### A.5 Radiotherapy treatments of animals with implants

The pig thigh with different implants was treated by 6 MV photon beams. The CMS XiO (version 4.40) (Computerized Medical System (CMS), St. Louis, MO) treatment planning system was used to produce treatment plans with different beam setups. The superposition convolution algorithm was used for the dose calculation. The electron‐density values were corrected properly in the treatment planning system calculation for steel and titanium.

Four treatment plans were designed: (1) a single‐field treatment (gantry angle: 0°, 200 MU), (2) two opposed fields (gantry angle: 0° and 180°, respectively, 100 MU/beam), (3) three fields (gantry angle: 0°, 120° and 240°, respectively, 66.7 MU/beam), and (4) three‐field intensity‐modulated radiation therapy (IMRT), in which the same gantry angles were used as in plan (3). In all cases, the target was the bone, and the isocenter was set at the center of the target. The source‐to‐axis distance (SAD) was 100 cm. For the IMRT plan, the doses were normalized to ensure 95% of the target volume receiving the prescribe dose of 200 cGy.

## III. RESULTS

### A.1 Monte Carlo simulation results

**Figure 3:**
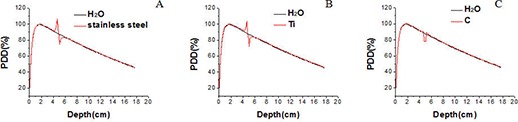
Percent depth dose curves without an implant and with different implants: (A) a stainless steel implant, (B) a titanium implant, and (C) a carbon fiber implant.

For a 10 cm × 10 cm photon field, the doses at the interface immediately above the implants increased by 20.1%, 16.7%, and 1.2% for the stainless steel implant, the titanium implant, and the carbon implant, respectively, compared to that in the water phantom in absence of the implants (Fig. 3). This dose increase can be explained by the enhanced backscattering from the high‐Z materials. The maximum dose difference was reduced to 1.9% at a point 0.3 cm above the implant and less than 1% at a point 0.4 cm above the implant since the backscattered electrons have very limited range.

The doses at the interface immediately below the implant decreased by 9.0%, 6.9%, and −2.2% for the stainless steel implant, the titanium implant, and the carbon implant, respectively. The dose difference was reduced to less than 1.5% at a point 1.2 cm below the stainless steel implant and at a point 0.9 cm below the titanium implant. This dose perturbation can be explained by the reduced electron fluence from the high‐Z materials compared to that from water (in absence of the metal implants). The second build‐up effect was significantly below the stainless steel implant and the titanium implant, while a slight dose increase was observed below the carbonaceous implant.

For a 3 cm × 3 cm photon field, the dose increase at the interface immediately above the implants was 19.9%, 16.5%, and −0.3% for the stainless steel implant, the titanium implant, and the carbon implant, respectively. The doses at the interface immediately below the implant decreased by 9.2%, 8.2%, and −1.4% for the stainless steel implant, the titanium implant, and the carbon implant, respectively — which were not significantly different from those for the 10 cm × 10 cm photon field.

### A.2 TLD measurement results in water

For a 10 cm × 10 cm photon field, the dose measured by the TLD immediately above the implant was 20.1%, 15.5%, and 0.1% higher for stainless steel, titanium plate, and carbon implants, respectively, than that without the implant, while the doses below the implant decreased by 8.5%, 7.8%, and −2.4%, respectively. The measurement results were consistent with the Monte Carlo simulation results within the combined measurement and calculation uncertainties of about 3%.

### A.3 Comparison of X‐ray radiographs and CT images of different implants

**Figure 4:**
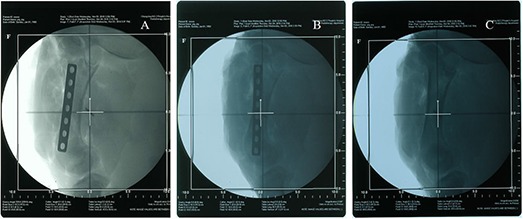
X‐ray radiographs of a stainless steel implant (A), a titanium implant (B), and a carbon implant (C).

**Figure 5:**
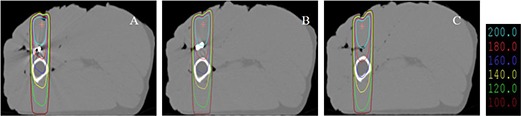
CT images of a stainless steel implant (A), a titanium implant (B), and a carbon implant (C) showing significant CT artifacts due to the metal implants. The isodose lines represent 200 cGy, 180 cGy, 160 cGy, 140 cGy, 120 cGy, and 100 cGy for a 6 MV photon beam.

The X‐ray radiographs of the three implants are shown in Fig. 4. The stainless steel and titanium alloy implants are clearly seen on the radiographs demonstrating their significant attenuation effects compared to the carbon fiber implant. On the CT image, the stainless steel implant exhibits more significant striking artifacts in the surrounding tissues than the titanium implant. The carbon fiber implant does not influence the CT image quality, as shown in Fig. 5.

### A.4 Comparison of radiotherapy treatment plans for different implants

The isodose distributions of a single 6 MV photon field for different implants are shown in Fig. 5. It is evident that the stainless steel implant has the greatest impact on the isodose distributions, followed by the titanium implant, and that the carbon fiber implant has hardly any influence. The differences in the external contours due to different implant insertions caused some ripples on the isodose lines, but significant dose perturbations occurred after the beam passing through the metal implants, demonstrating more photon attenuation by the stainless steel implant than by the titanium implant. The isodose distributions were consistent before and after the carbon fiber implantation. The results are in accordance with the Monte Carlo simulation results.

**Figure 6:**
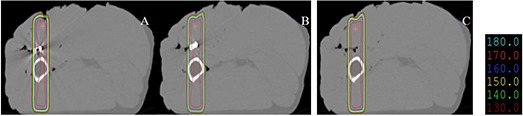
Isodose distributions of two opposed 6 MV photon fields for a stainless steel implant (A), a titanium implant (B), and a carbon implant (C). The isodose lines represent 180 cGy, 170 cGy, 160 cGy, 150 cGy, 140 cGy, and 130 cGy for a 6 MV photon beam.

The isodose distributions of two opposed 6 MV photon fields for different implants are shown in Fig. 6. The greatest dose perturbation resulted from the stainless steel implant; the 170 cGy isodose line for the stainless steel implant differed significantly from that for the carbon fiber implant. The titanium implant also showed some effects on the isodose distributions

**Figure 7:**
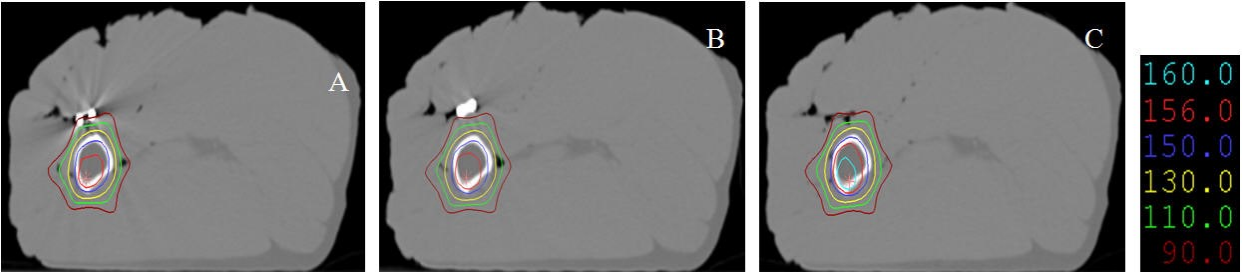
Treatment plans for different implants irradiated by three 6 MV photon fields incident at 0°, 120°, and 240° gantry angles. The effects of the metal implants were much reduced, since only one beam had gone through the metal implants.

Figure 7 shows the treatment plans for different implants irradiated by three 6 MV photon fields incident at 0°, 120°, and 240° gantry angles. The effects of the metal implants were much reduced, since only one beam had gone through the metal implants.

**Figure 8:**
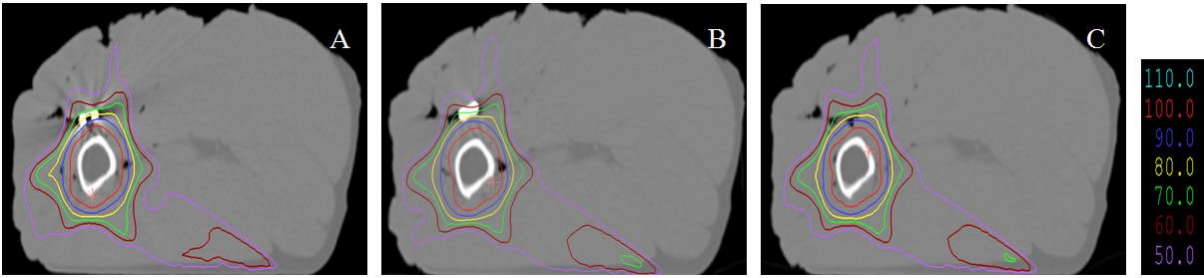
Isodose distributions of three intensity‐modulated 6 MV photon fields incident at 0°, 120°, and 240° gantry angles for a stainless steel implant (A), a titanium implant (B), and a carbon implant (C). The isodose lines represent 110%, 100%, 90%, 80%, 70%, 60%, and 50% of the prescription dose of 200 cGy.

Figure 8 shows the isodose distributions for different implants irradiated by three intensity‐modulated 6 MV photon fields. Similar to the results in Fig. 7, only small dose perturbations were observed for metal implants compared to the carbon fiber implant. The MU ratio for the stainless steel implant, the titanium implant, and the carbon fiber implant (to achieve the same target dose of 200 cGy) was 1.11:1.04:1.00.

## IV. DISCUSSION

In this study, we evaluated the dosimetric properties of carbon fiber implants by comparison with the dosimetric properties of stainless steel and titanium implants. It is shown by X‐ray radiographs and CT images that metal implants can cause more X‐ray attenuation and CT imaging artifacts that can further affect the dose calculation either using commercial treatment planning systems or using Monte Carlo simulations. For 6 MV photon beams, the dose perturbation effects are more severe for a stainless steel implant than for a titanium implant, while a carbon fiber implant has almost no effect on the dose distribution. Significant differences in dose between the planning system and Monte Carlo in the high‐Z region were observed, possibly due to the loss of electron equilibrium.^(^ ^13^ ^)^ In the present study, the superposition convolution results were consistent with the Monte Carlo results; significant dose perturbations occurred in both in areas near the metal implants. The TLD measurements confirmed the Monte Carlo calculation results. The radiation dose impact of the implants was basically the same for 6 MV photon beams with field sizes between 3 cm × 3 cm and 10 cm × 10 cm. Greatest dose increase in front of the implant and greatest dose reduction behind the implant were observed when a single 6 MV photon beam passed through the stainless steel implant. These perturbation effects were much reduced for two opposed fields and further reduced for three fields when more beams avoided the implants. Therefore, multifield treatments are more advantageous than single or opposed‐field treatments for metal implants. However, conformal avoidance may be limited for some target locations. Intensity‐modulated radiation therapy makes it possible to obtain a better dose distribution after the implantation of metal implants at the cost of increased MUs, which will increase the dose to the normal tissues and also prolong the treatment time. Carbon fiber implants, in comparison, can be a better alternative for patients who will receive radiotherapy after their orthopedic implantation.

In this work, we only investigated the dose perturbation effects of the three orthopedic implants using 6 MV photon beams. Both the forward and backscattered dose perturbation effects of high‐Z materials are greater for higher photon energies that are used in radiation therapy^(^ ^14^ ^)^ and, therefore, they are less ideal as implant materials for these high‐energy photon beams as compared to carbonaceous materials. Furthermore, photon energies higher than 10 MV are not often used in IMRT treatments due to their neutron components that can be more detrimental to younger radiotherapy patients. Therefore, the advantages of carbon fiber implants over metal implants would be greater for conventional radiotherapy treatments with fewer high‐energy photon beams.

## V. CONCLUSIONS

In summary, carbon fiber implants have minimal dose perturbation effects compared to commonly‐used metal implants, and are more suitable for radiotherapy patients with orthopedic implants, in terms of clinical dosimetry.

## ACKNOWLEDGMENTS

The authors would like to acknowledge Dr. Lili Chen and Dr. C‐M Charlie Ma of Fox Chase Cancer Center for technical assistance. Deep gratitude also goes to Siemens Medical Solutions, Inc. for the provision of the accelerator data. This work was funded by the municipal social development project of the Changzhou City, Jiangsu Province, China (CS20102019).
